# Imaging features of foreign body granuloma in the lower extremities mimicking a soft tissue neoplasm

**DOI:** 10.1080/03009730802602455

**Published:** 2009-02-04

**Authors:** Akira Ando, Masahito Hatori, Yoshihiro Hagiwara, Shuji Isefuku, Eiji Itoi

**Affiliations:** ^1^Department of Orthopaedic Surgery, Tohoku University Graduate School of MedicineSendaiJapan; ^2^Department of Orthopaedic Surgery, Sendai Medical CenterSendaiJapan

**Keywords:** Extremities, foreign body, granuloma, MRI, wooden splinter

## Abstract

Foreign body granuloma is a tissue reaction for retained foreign bodies after skin-penetrating trauma. Detection of retained foreign bodies can be extremely difficult when the patients present with non-specific symptoms such as pain and/or swelling without recognizing a previous trauma. We report three patients of foreign body granulomas in the lower extremities with emphasis placed on their unique clinical and radiological features. The involved sites were the foot, posterior thigh, and posterior lower leg, with wooden splinters in two patients and a fragment of tile in one. Plain radiographs could not reveal the existence of foreign bodies. Magnetic resonance imaging (MRI) showed foreign bodies as low intensities on both T1- and T2-weighted images in two patients, and the surrounding reactive lesion as low to iso intensities on T1- and high intensities on T2-weighted images in all the patients. The peripheral areas of the lesion were strongly enhanced after gadolinium injection. Ultrasound sonography could clearly visualize a foreign body as an echogenic area with posterior acoustic shadowing in one patient. The surrounding ring-like reactive lesion is easily mistaken for a soft tissue neoplasm when foreign bodies are not identified. The key to arriving at the correct diagnosis is to clarify the previous trauma and to identify foreign bodies with low signal intensities on both T1- and T2-weighted images and/or the characteristic ring-like enhancement on MRI. It is also necessary to rule out a foreign body granuloma whenever we see patients with a soft tissue tumor in the extremities, irrespective of their previous trauma history.

## Introduction

Foreign body granuloma is mainly divided into iatrogenic gossypiboma by retained surgical sponge during operation (1) and granulation by a penetrating foreign body such as wooden splinter or other materials (2). If a history of antecedent skin-penetrating trauma or previous operation is recognized, it is important to take foreign body granuloma into consideration as a differential diagnosis from soft tissue tumors. However, in cases where the history of trauma is uncertain, and moreover the patients present for evaluation several months or even years after initial injury, the radiological appearance of foreign body granuloma can be confusing and can even mimic a neoplasm (3). There have been few reports to describe overall radiological imaging features including computed tomography (CT), magnetic resonance imaging (MRI), and/or sonography of foreign body granulomas (4). We present three patients with foreign body granulomas mimicking soft tissue tumors in the lower extremities, with emphasis placed on their unique clinical and imaging features of CT, MRI, and/or sonography, and describe how to arrive at the correct diagnosis.

## Case reports

### Case 1

**Figure 1.**
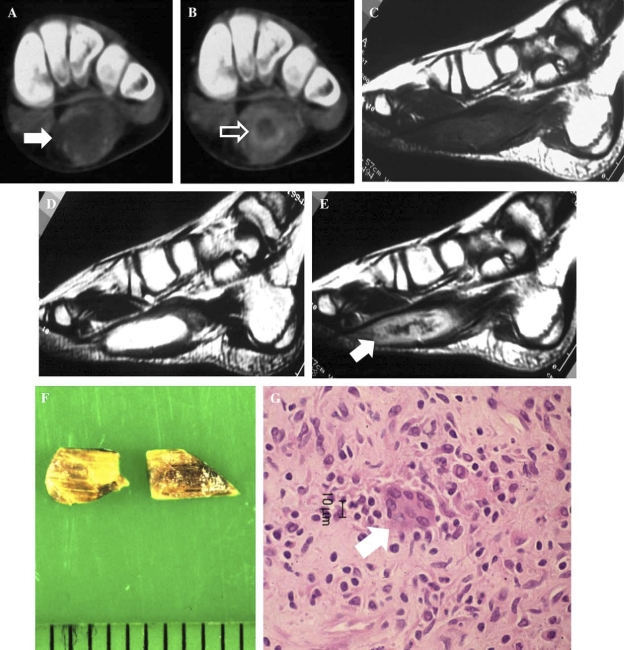
Nine-year-old girl with foreign body granuloma of the left foot. A and B: Coronal plain and enhanced computed tomography (CT) of the left foot. Plain CT showed a mass (solid arrow) with iso to low-density area. Peripheral area of the lesion was enhanced, but the center of the lesion was not (open arrow). C and D: Sagittal T1- and T2-weighted images of the left foot. The mass had iso intensities on T1- and high intensities on T2-weighted images to the muscles. Foreign bodies were not identified. E: After gadolinium injection, the peripheral area (arrow) of the lesion was strongly enhanced. F: Two wooden splinters (5×4 mm) were removed at surgery. G: Histological evaluation of the wall of the mass. Inflammatory cell proliferation with giant cell formation (arrow) was observed.

A 9-year-old girl was referred to our institution with a 1-year history of mass and pain in her left foot. She had suffered skin-penetrating trauma by wood 2 years earlier, but the history of foreign body penetration was not obtained at the time of the initial interview. Physical examination showed a 6×3 cm soft tissue mass in the plantar part of her left foot. Hematological investigations were unremarkable with normal blood cell count and normal biochemistry. Plain radiographs showed a soft tissue mass with no apparent calcifications or foreign bodies. CT showed an iso to low-density mass at the plantar aspect of her foot (Figure 1A). The peripheral area of the lesion was enhanced, but the center was not (Figure 1B). MRI showed the mass as iso signal intensities on T1-weighted images (Figure 1C) and high signal intensities on T2-weighted images (Figure 1D) to the muscles. The peripheral area of the lesion was strongly enhanced after gadolinium injection (Figure 1E). We could not diagnose at that time, and differential diagnoses were a liquefied hematoma, a cold abscess, or cystic degeneration within a soft tissue sarcoma. Surgery revealed two wooden foreign bodies (5×4 mm) surrounded by granulation tissue (Figure 1F). Histological examination showed inflammatory cell proliferation with giant cell formation, which was coincident with the findings of foreign body granuloma (Figure 1G).

### Case 2

**Figure 2.**
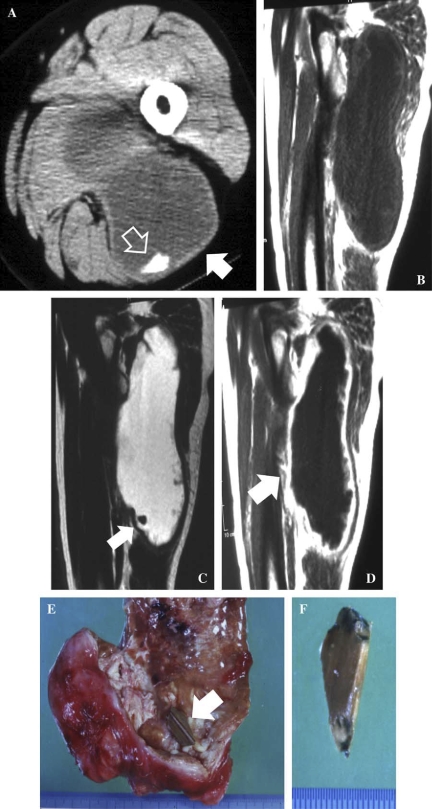
Fifty-five-year-old woman with foreign body granuloma of the left posterior thigh. A: Axial computed tomography (CT) of the left thigh. Huge mass in the hamstring muscle (solid arrow) with a small high-density fragment (open arrow) was observed. B and C: Sagittal T1- and T2-weighted images of the left posterior thigh. The mass had low intensities on T1- and high intensities on T2-weighted images to the muscles. The small fragment inside the mass showed low intensities to the muscles (arrow). D: The peripheral area of the lesion (arrow) was strongly enhanced after gadolinium injection. E: Cut surface of the lesion. The mass was filled with much fluid and granulation tissue. A foreign body (arrow) was seen inside the mass. F: A fragment of tile (3×1 cm) was removed at surgery.

A 56-year-old woman presented with a growing mass in the left posterior thigh after trauma. She had fallen from upstairs and had treatment for a right rib fracture and hemothorax 4 years prior to her presentation. On physical examination a 20×15 cm mass in the left posterior thigh was noted. The blood cell count, biochemistry, and erythrocyte sedimentation rate were all within the normal range. Plain radiographs showed a soft tissue mass, but did not reveal the retained foreign body. CT showed a huge mass in the hamstring muscles (Figure 2A). The high-density small fragment within the mass was clearly visible. The wall of the mass was iso density and the area inside the mass showed low density to the muscles. MRI showed the mass as low intensities on T1- and high intensities on T2-weighted images to the muscles (Figure 2B, C). The small fragment inside the mass showed low intensities to the muscles on both T1- and T2-weighted images. After gadolinium injection, the wall of the mass was strongly enhanced (Figure 2D). A fragment of tile (3×1 cm) was found inside a cystic tumor lined with granulation tissue (Figure 2E, F).

### Case 3

**Figure 3.**
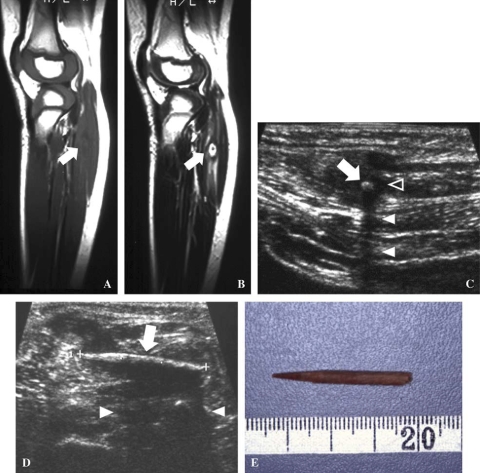
Three-year-old girl with foreign body granuloma of the right posterior lower leg. A and B: Sagittal T1- and T2-weighted images of the right posterior lower leg. The mass had low intensities on T1- and high intensities on T2-weighted images (arrows). Central area of the lesion had low intensities both on T1- and T2-weighted images. C and D: Sonography imaging perpendicular and horizontal to the foreign body. The foreign body was observed as a hyperechoic lesion (arrow) with posterior acoustic shadowing (solid arrowheads) and hypoechoic halo (open arrowhead). E: A 3-cm long tip of toothpick was removed at surgery.

A 3-year-old girl was referred to our institution with 1-week lasting pain and limping after penetrating trauma by a toothpick. Her parents were not certain whether the toothpick was removed successfully at the injury. Physical examination revealed a painful 20×15 mm mass in the right proximal posterior lower leg. The results of hematologic and chemical studies were normal. Conventional radiography did not reveal any calcifications or foreign bodies in the soft tissue of the lower leg. The mass had iso intensities on T1- and high intensities on T2-weighted images. The small lesion inside the mass showed low intensities on both T1- and T2-weighted images (Figure 3A, B). It looked like a ‘target appearance’ especially on T2-weighted images. Ultrasound sonography visualized a foreign body as a hyperechoic focus with posterior acoustic shadowing and a hypoechoic halo (Figure 3C, D). At surgery, a 3-cm-long toothpick tip was removed (Figure 3E).

## Discussion

It is very difficult to suspect foreign body granuloma particularly when the patient or the patient's family did not pay attention to a previous trauma. The morphology of the lesions varies widely depending on the type of the material involved, the size of the foreign body, and the site of trauma. When the history of the previous trauma is not obtained and the foreign body inside the lesion is not recognized radiologically, foreign body granuloma is easily mistaken for a primary soft tissue neoplasm (2). In the first patient we could not find the foreign body, and the differential diagnoses were a liquefied hematoma, a cold abscess, and cystic degeneration within a soft tissue sarcoma. Even when the history of previous trauma is not obtained, to suspect the existence of a foreign body is of importance to narrow the differential diagnosis, because the extremities are a common location for retained foreign bodies. In other words, a foreign body granuloma should be ruled out whenever we see patients with a soft tissue tumor in the extremities, irrespective of their previous trauma history.

Plain radiography should be taken when the existence of foreign bodies is suspected. If the foreign body is radiopaque, we can easily identify the foreign body. However, to identify radiolucent foreign bodies, such as wooden splinters, is extremely difficult by plain radiographs. Plain radiography has been reported to reveal a wooden foreign body in only 15% of patients (5). We could not detect the foreign bodies in any of the patients because the size of the foreign body was not sufficient to create radiolucency.

CT has been reported to be useful for identifying foreign bodies (6). The appearance of foreign bodies on CT varies from a low-density area in acute cases to a high-density area in chronically retained cases. When a dry foreign body enters the human body, it is filled with air. After that, the foreign body absorbs the surrounding exudate and increases its density (4). We could clearly visualize a foreign body in the second patient as a high-density area, but we could not identify a foreign body in the first patient perhaps due to the small size of the body. The values of density for smaller objects may vary relating to partial volume averaging, and moreover different types of foreign bodies have variable density (4). Surrounding reactive lesions are shown as low to iso density areas to the muscles, but as expected the detection of these lesions and their extent is better delineated with MRI than CT (6). CT is superior to MRI to identify radiopaque foreign bodies but does not have advantage for detection of radiolucent foreign bodies and visualization of the surrounding reactive lesion.

MRI shows the foreign bodies as low signal or signal void to the muscles on both T1- and T2-weighted images (4,6). However, the identification of foreign bodies is difficult on MRI when the bodies are small. We could not make a diagnosis of foreign body granuloma before surgery in the first patient because the size of the foreign bodies was too small to identify, and we did not suspect their existence. When a foreign body is surrounded by inflammatory tissues or a hematoma, a ring of low signal on T1- and high signal on T2-weighted images around the foreign bodies is observed demonstrating a target appearance (7). In the third patient, a target appearance was clearly visible on both T1- and T2-weighted images, and we could strongly consider the existence of a foreign body. The surrounding reactive lesion is easily mistaken for a soft tissue neoplasm when a foreign body is not identified because the peripheral area of the lesion is strongly enhanced by gadolinium (4). The central area of the reactive lesion is observed as low intensities on T1- and high intensities on T2-weighted images with no enhancement, which suggests that the lesion is myxomatous or cystic (8,9). The key to diagnose correctly on MRI is identification of a low signal or signal void lesion inside the mass and the surrounding ring-like reactive lesion.

If there is concern about the existence of foreign bodies, ultrasound sonography should be used. Sonography has been studied for detection and localization of foreign bodies and proved to be sensitive and specific (10,11). Given the markedly different acoustic impedance of foreign bodies, such as wood and soft tissues, they are easily identified as echogenic ones with marked posterior acoustic shadowing. Foreign bodies are also surrounded by hypoechoic halos which consist of reactive lesions such as hematoma, edema, and granulation tissue (10). We could clearly visualize a foreign body in the third patient with posterior acoustic shadowing and a hypoechoic halo. When compared with MRI and CT, ultrasound sonography is less expensive, readily available, and superior in the detection of foreign bodies. Sonography is a modality which should be adopted when patients present with a history of antecedent skin-penetrating trauma or when foreign body granuloma is suspected from MRI or CT (4).

In conclusion, the human body, especially the foot and hand, is inevitably exposed to skin-penetrating trauma. When foreign body granulomas are suspected, identification of low signal or signal void foreign bodies with a characteristic ring-like reactive lesion on MRI, and hyperechoic lesions with posterior acoustic shadowing on sonography, is important for correct diagnosis.
